# Primary and Secondary Symptoms

**Published:** 1891-03

**Authors:** M. W. Vandenburg

**Affiliations:** Fort Edward, N. Y.


### PRIMARY AND SECONDARY SYMPTOMS.

M. W. Vandenburg, A. M., M. D., Fort Edward, N. Y.

After reading and re-reading Dr. Gilbert's article, in the February number of The Homœopathic Physician, I find myself more and more in doubt how to apply it to the study of our materia medica.

It is fully allowed that there are primary and secondary symptoms, and it is claimed that " the latter are the bane of our materia medica," " and that a homœopathic prescription cannot be based upon secondary symptoms."

To one who is anxious to escape this "bane," the question naturally arises, Where is the materia medica that differentiates the primary and secondary symptoms?

Is it then impossible to make a homœopathic prescription from Hahnemann, or Hering, or Allen, or Lilienthal, or Cowperthwait? Have all these years passed, and no one yet been able to make a genuine homœopathic prescription?

If now " primary symptoms be those first appearing as the result of the action of a medicinal substance upon any tissue," and if "the secondary symptoms must be the result of reaction, which must, and can only come from the system," and if also, " the last symptoms to appear are the most characteristic; not the so-called secondary symptoms, but those which, while they come late, are the first evidence of an attack upon the particular part of the system," who shall be able to assuredly separate the one from the other, and give us a materia medica without a " bane "?

Again, it is stated, "the high attenuations produce upon the [healthy] system symptoms like those produced by the vital force in reacting against the crude doses."

The bracket is my own, but this is what it means as I suppose.

If this be the case, then either the provings with crude doses are not reliable, or at least not available for homœopathic use, or, per contra, the provings from high attenuations are useless.

For, if secondary symptoms of crude doses are "the bane of materia medica," the primary symptoms of high attenuations, being the same, must also be a bane.

Or, on the other hand, if the provings of crude doses are untrustworthy in their primary symptoms, then surely their secondary symptoms are the real materia medica, since "these are like those produced by the higher attenuations upon the healthy system."

It is then the secondary symptoms of the higher attenuations that are the real bane against which we are to guard.

I have tried to follow the doctor candidly, considerately, and with perfect fairness, and this is where he has brought me.

It may be Dr. Gilbert denies the value of primary symptoms from crude doses; but to be consistent he must allow the value of their secondary symptoms, those "like the primary of the higher attenuations."

If this is the case what becomes of conclusion No. 1—"Primary symptoms" only are indications for the selection of the remedy, as taught by Hahnemann?

So also of No. 2.—"There are no secondary symptoms of a remedy, but such so-called symptoms are the evidences of the reaction of the system."

No. 3 reads, "remedies follow each other, homœopathically, in which the action of the second (primary action) is similar to the reaction (secondary action) against (of) the first."

Here then is a wide field for this "bane" to become very useful. Again I ask, is it that these "secondary symptoms" of the "higher attenuations" are so baneful.

No. 4 reads, "the dose must be reduced below the sick-making power (that is, the one capable of producing a crudedrug primary symptom, if we rightly understand his meaning), until it is capable of inducing an action in an opposite direction to the effect of the crude drug upon the well (that is, a secondary symptom of the crude drug), without any appreciable aggravation, and this constitutes a 'high attenuation.'"

Here again is evident the value of the "secondary symptom" of the crude drug.

No. 5 reads, "the dose for the sick must be smaller (higher) than that required to produce the required reaction in the well."

This is very obscure; it would seem to be a repetition of the preceding point. The dose must be so small that it would not produce a secondary symptom if administered to the well, like the one to be cured in the sick.

Here, then, it seems again, we are to be guided wholly by the secondary symptoms of crude doses, in finding our simillimum, and not by the primary ones.

Altogether we are getting more and more deeply involved. We must stop while we can. One question more I would like to ask. Does the doctor reject the proving of "the higher attenuations," those so high that they are "capable of inducing an action in an opposite direction to the effect of the crude drug"?

And what would he have done with the "secondary effects of such provings as the 30th, advised by Hahnemann and Carroll Dunham"?

What would he have done with the primary symptoms of crude doses?

How would he invariably distinguish primary from secondary symptoms?

Are not the secondary symptoms in any case as much peculiar to the drug as the primary, and if not, why?

---

KOCH'S LYMPH.—Dr. Samuel Swan, No. 13 West 38th St., New York, authorizes us to say that he will furnish grafts of Koch's lymph in the two-hundredth potency, *free* to those who wish to prove it as a remedy.

